# Identification of multiple physicochemical and structural properties associated with soluble expression of eukaryotic proteins in cell-free bacterial extracts

**DOI:** 10.3389/fmicb.2014.00295

**Published:** 2014-06-20

**Authors:** Alexander A. Tokmakov

**Affiliations:** Research Center for Environmental Genomics, Kobe UniversityKobe, Japan

**Keywords:** cell-free protein synthesis, protein solubility, physicochemical and structural protein properties, categorical data analysis, correlation analysis

## Abstract

Bacterial extracts are widely used to synthesize recombinant proteins. Vast data volumes have been accumulated in cell-free expression databases, covering a whole range of existing proteins. It makes possible comprehensive bioinformatics analysis and identification of multiple features associated with protein solubility and aggregation. In the present paper, an approach to identify the multiple physicochemical and structural properties of amino acid sequences associated with soluble expression of eukaryotic proteins in cell-free bacterial extracts is presented. The method includes: (1) categorical assessment of expression data; (2) calculation and prediction of multiple properties of expressed sequences; (3) correlation of the individual properties with the expression scores; and (4) evaluation of statistical significance of the observed correlations. Using this method, a number of significant correlations between calculated and predicted properties of amino acid sequences and their propensity for soluble cell-free expression have been revealed.

## INTRODUCTION

Heterologous protein synthesis is widely used for production of recombinant proteins. Particularly, eukaryotic proteins and their domains are often expressed in bacterial hosts (Yokoyama, 2003; Sorensen and Mortensen, 2005; Sivashanmugam et al., 2009; Chen, 2012). However, only a minor fraction of all proteins can be successively produced in bacterial host systems. Presently, the factors determining expression success in these systems are poorly understood. Various physicochemical features of an amino acid sequence have been implicated as determining factors of soluble protein expression in bacteria (Bertone et al., 2001; Dyson et al., 2004; Goh et al., 2004; Idicula-Thomas and Balaji, 2005).

Recently, cell-free systems of protein synthesis have been developed that offer numerous advantages over cell-based expression (reviewed in Spirin, 2004; Katzen et al., 2005; He, 2008). The cell-free systems allow genome-scale expression of various amino acid sequences under strictly controlled uniform conditions. The productivity of bacterial cell-free synthesis reaches several milligrams of protein per milliliter of reaction mixture (Kigawa et al., 1999). Most often, the purpose of heterologous cell-free synthesis is to produce properly folded and functionally active protein product in the amounts sufficient for structural and functional studies. However, the folding of eukaryotic proteins is greatly compromised in bacterial extracts due to intrinsic differences between the cytoplasmic environments of prokaryotic and eukaryotic cells. Moreover, many eukaryotic proteins require multiple post-translational modifications (PTMs) to attain a native, biologically active state. However, the bacterial expression systems have only a limited capacity for PTMs.

In the present paper, we describe an approach aimed at identification of numerous physicochemical, structural and functional properties of amino acid sequences, including the sites of multiple PTMs, associated with soluble expression of eukaryotic proteins in bacterial cell-free extracts, and highlight major correlations obtained using this approach.

## METHOD

### METHOD OVERVIEW

The developed method is intended for analysis of output from an existing cell-free protein production pipeline. Thus, this paper does not cover the experimental workflow of protein production. It is described in detail in the previous publications (Yabuki et al., 2007; Kigawa et al., 2008; Kurotani et al., 2010; Tokmakov et al., 2012). Here, the focus is set on the processing of experimental data with the purpose of identification of multiple physicochemical and structural properties associated with soluble expression of eukaryotic proteins in cell-free bacterial extracts. Important for the developed approach is that all the proteins in the analyzed dataset are expressed under the same uniform set of conditions. This minimizes the influence of sequence-independent factors and makes possible adequate categorical assessment of expression data (see Categorical Assessment of Expression Data section). The affinity purification tags should be avoided in the expressed sequences because they hinder the analysis of expression correlations by decreasing the role of sequence-specific determinants.

**FIGURE 1:**
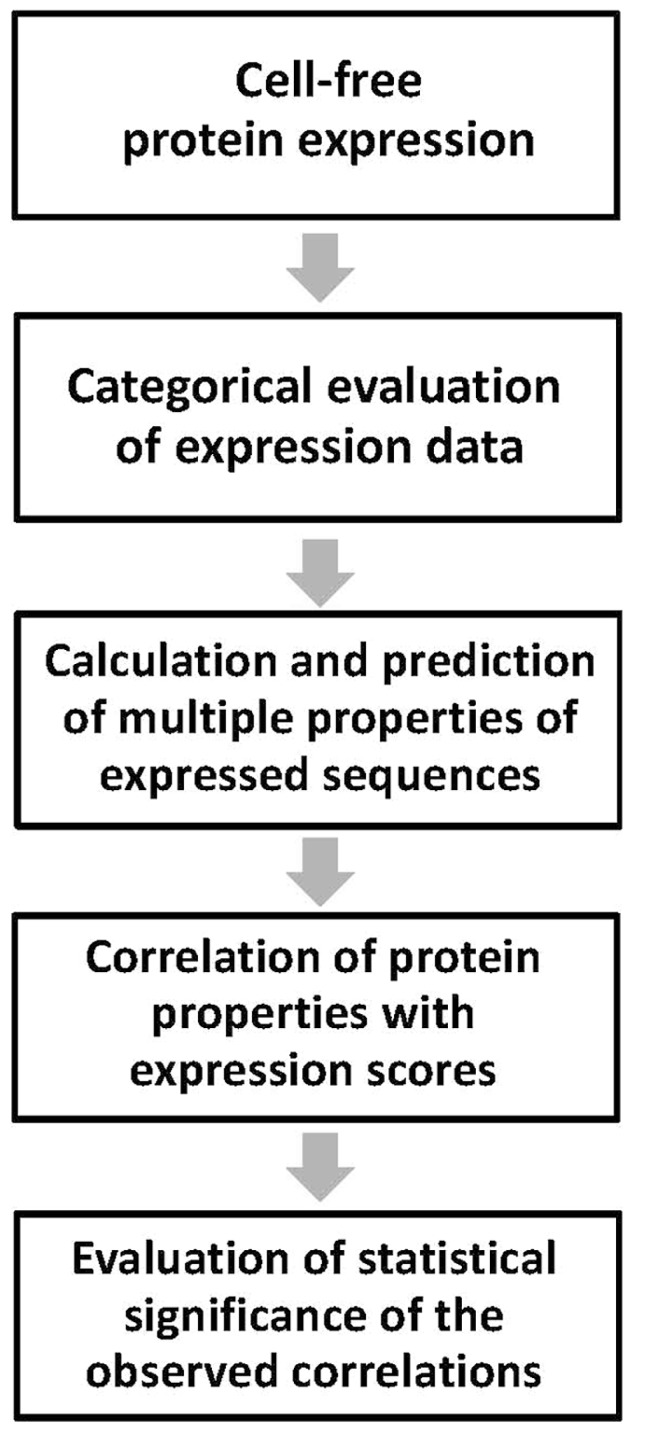
**Workflow of the analysis.** Main steps of the experimental data processing are presented. Cell-free protein expression should be performed under the same uniform set of conditions.

The main steps of the proposed method are summarized in **Figure 1**. They include: (1) categorical assessment of the experimental results of protein expression; (2) determination of multiple physicochemical and structural properties of the expressed amino acid sequences using computational and predictive bioinformatics tools; (3) correlation of the individual protein properties with the experimental expression scores; and (4) evaluation of statistical significance of the observed correlations. The developed approach has been extensively used to analyze experimental expression of human proteins and their domains in *Escherichia coli* bacterial extracts (Kurotani et al., 2010; Tokmakov et al., 2012; see Results and Discussion section). However, it can be universally applied to any other cell-free system of heterologous protein synthesis. Each step of the above protocol is detailed below.

### CATEGORICAL ASSESSMENT OF EXPRESSION DATA

**FIGURE 2:**
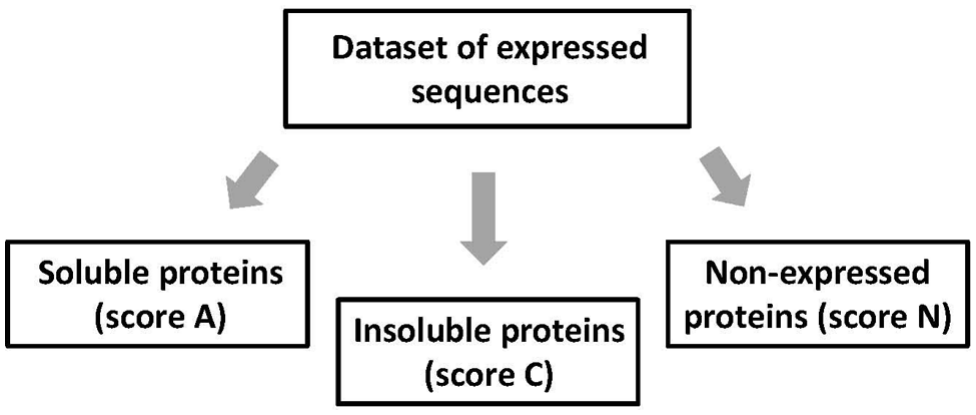
**Categorical evaluation of protein expression data.** At this stage, all expressed sequences are categorized into the three mutually exclusive categories – soluble (A), insoluble (C), and non-expressed (N) proteins.

At the stage of expression assessment, all studied proteins are classified into three mutually exclusive categories – soluble (A), insoluble (C), and non-expressed (N) proteins (**Figure 2**). Each sequence can only be placed into one expression category and not into another. Soluble and insoluble products of protein synthetic reaction can be separated by centrifugation at 10,000 × *g* for 10 min and visualized by Coomassie Blue staining after SDS PAGE. The scores A, C, and N are assigned as follows: A, soluble proteins expressed at the level of more than 0.1 mg per ml of cell-free extract; C, expressed, but insoluble proteins; and N, non-expressed proteins with the expression level below 0.1 mg/ml. The protein products expressed at the level below 0.1 mg/ml are difficult to visualize on the Coomassie-stained gels, because the specific protein bands are masked by the endogenous proteins of the bacterial extract. Proteins that are expressed at a lower than expected molecular size should be classified into the category N, as they cannot attain proper structure and function. Notably, in this setting, the score A provides the upper estimation of soluble protein expression, because the procedure of centrifugation at 10,000 × *g* cannot discriminate between small protein aggregates and truly soluble proteins. Often, expressed proteins can be found in both soluble and insoluble fractions of the bacterial extract. Lane-to-lane comparison of total and supernatant fractions of the extract in PAGE gels is usually sufficient to establish the preferential pattern of protein expression.

### CALCULATION AND PREDICTION OF MULTIPLE PROPERTIES OF EXPRESSED SEQUENCES

**FIGURE 3:**
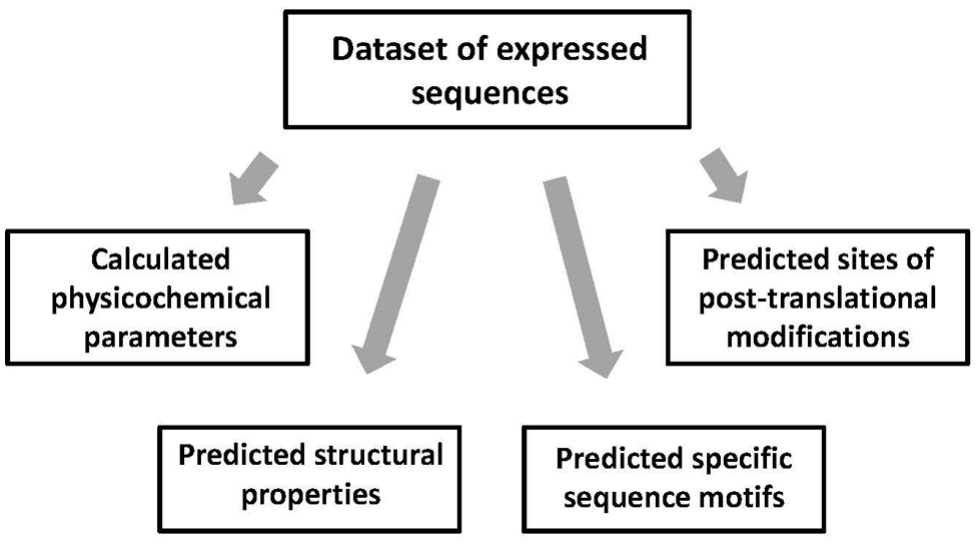
**Calculated and predicted features of expressed sequences.** Various parameters and properties of the amino acid sequences in the expression dataset can be classified into four major types. Protein features are calculated or predicted using existing bioinformatics algorithms and tools.

In this step, multiple features of the amino acid sequences in the expression dataset are calculated or predicted using existing bioinformatics tools. Various protein properties can be classified into the four major types, including physicochemical parameters, structural properties, the presence of specific sequence motifs, and the presence of PTM sites (**Figure 3**). Many of the physicochemical parameters, such as protein length, molecular weight, amino acid composition, number of charged residues, pI, hydrophobicity, etc., can be calculated using the free ProtParam tool available at the Expasy server^1^. On the other hand, it is difficult to precisely calculate high-dimensional protein properties, because the 3D structures of expressed protein targets are usually unknown. Still, it is possible to deduce some structural features of the proteins in the expression dataset using existing prediction algorithms. Admittedly, some of these algorithms have quite low prediction accuracy, not exceeding 80%. The low accuracy of prediction thwarts the following correlation analysis, making impossible detection of weak correlations. Solvent accessibility can be assessed with the ACCpro 4.0 software downloaded from the SCRATCH Protein Predictor server (Cheng et al., 2005^2^) and content of secondary structure is evaluated with the PREDATOR 2.1.2 tool (Frishman and Argos, 1997) provided online^3^. Coiled coil structures are predicted with the pepcoil tool provided online^4^ (Lupas et al., 1991) and content of disordered structure is predicted with the RONN software (Yang et al., 2005^5^). The specific sequence motifs in proteins can also be predicted using available bioinformatics tools. PEST regions, signal sequences, and transmembrane domains are predicted with the tools provided online^6^^,^^7,8^. The sites of multiple PTMs, such as phosphorylation, glycosylation, amidation, Asx hydroxylation, sulfation, prenylation, etc., can be predicted using the PROSITE scanning tool PS_SCAN available online at http://www.hpa-bioinfotools.org.uk/cgi-bin/ps_scan/ps_scanCGI.pl. The sites of ubiquitination and SUMOylation are predicted using the site-specific predictors UbPred (Radivojac et al., 2010) and SUMOsp 2.0 (Ren et al., 2009) freely downloadable for academic research from http://ubpred.org/ and http://sumosp.biocuckoo.org/, respectively. The sites of S-palmitoylation are predicted with the CSS-Palm tool (Ren et al., 2008^9^) and S–S bonds can be predicted using the DIpro tool (Cheng et al., 2006) downloadable free from http://download.igb.uci.edu/intro.html.

### CORRELATION OF THE INDIVIDUAL PROPERTIES WITH EXPRESSION SCORES

**FIGURE 4:**
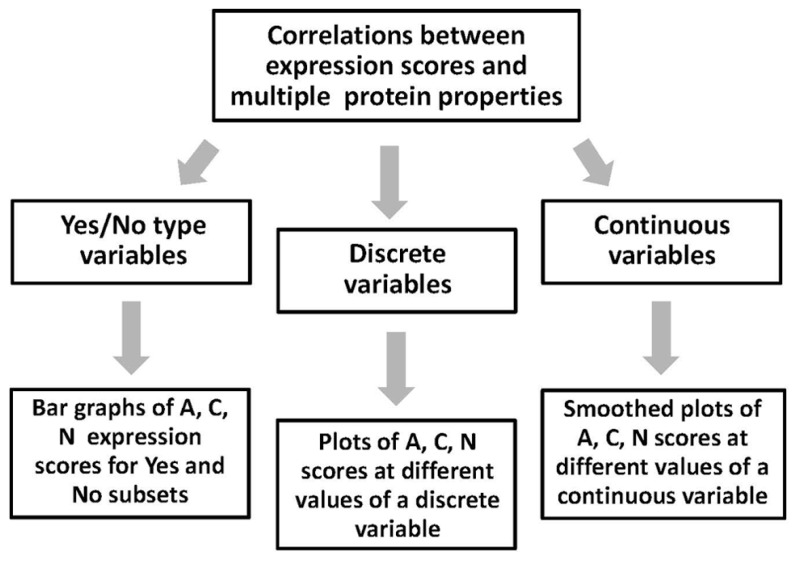
**Correlation of expression scores with multiple protein features.** Processing and presentation of correlation data depends on the type of analyzed features (variables). The three types of the features processed by this analysis include yes/no, discrete, and continuous variables.

The multiple protein properties calculated and predicted using the above bioinformatics tools can be categorized into the three types, including yes/no, discrete, and continuous variables (**Figure 4**). Data processing and presentation differs for the three types of variables. The yes/no type variables, such as single-event PTMs, are the features that can be either present in or absent from proteins. To present the expression data associated with these variables, the bar graphs can be built, which show the ratio of proteins in the expression categories A, C, and N. The graphs should represent two subsets of proteins, excluding and including the analyzed feature. Total number of sequences in the two subsets should be defined. Using these graphs, it is easy to make a side-by-side comparison of the data for the two subsets and deduce the tendencies in protein expression amenability associated with the analyzed feature. To present the expression correlations associated with the discrete variables related to the protein futures repeatedly observed in the analyzed sequences, such as abundant multi-site PTMs, another type of data presentation is more convenient. In this case, the percentage of proteins in the expression categories A, C, and N is plotted at different values of analyzed parameter, covering the entire parameter range in the dataset. In addition, the distribution of dataset proteins according to parameter values should be presented. The distribution graphs provide important information concerning the abundance of studied protein features in the analyzed dataset. The processing of data associated with continuous variables, such as sequence hydrophobicity, solvent accessibility, content of intrinsic disorder, etc., is similar to that described for discrete variables. The graphs of A, C, and N scores, as well as the distribution graphs should be provided in the full range of continuous feature values. Curve smoothing is recommended to straighten the graphs obtained with continuous variables. It can be performed using the Excel chart smoothing algorithm. The examples of data presentation for the three types of variables associated with different protein properties are provided in our recent publication (Tokmakov et al., 2014).

### STATISTICAL SIGNIFICANCE OF THE OBSERVED CORRELATIONS

**FIGURE 5:**
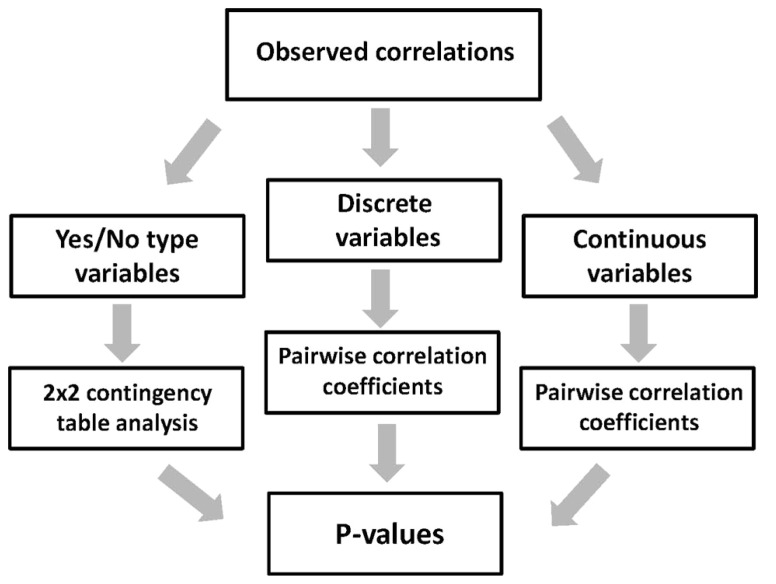
**Evaluation of statistical significance of the observed correlations.** Method for statistical evaluation of correlation data is chosen according to the type of analyzed protein features (variables). The three types of the features processed by this analysis include yes/no, discrete, and continuous variables.

The expression data processed by the proposed method represent categorical datasets, where all expressed sequences are classified into three categories – soluble (A), insoluble (C), and non-expressed targets (**Figure 2**). Thus, to evaluate the statistical significance of the observed correlations between the multiple protein features and protein amenability to cell-free expression, the categorical data analysis should be applied (Xu et al., 2010). The estimation of statistical significance should be provided for each expression category (A, C, and N). In addition, multiple protein properties are also categorized into the three types, such as yes/no, discrete, and continuous variables (**Figure 4**). Evaluation of statistical significance differs for the three types of variables. To deduce the statistical differences associated with yes/no type variables, the two-way contingency table test can be applied (**Figure 5**). The Fisher’s exact *p*-values can be computed using the tool provided on line at http://statpages.org/ctab2x2.html. Usually, a confidence level of 95% is set up as the null hypothesis rejection threshold. To evaluate the statistical significance of expression correlations associated with the discrete variables, which have a finite number of possible values, as well as the continuous variables, Pearson’s pairwise correlation coefficients should be calculated (**Figure 5**). The percentage of proteins in the expression categories A, C, and N should be paired with the values of the analyzed variable in the full range of variable values observed in the dataset. Statistical significance of the correlation coefficients is validated by calculating one-tailed probability values, given the value of correlation coefficient (*r*) and the sample size (*n*), with the significance level set to 0.05. Calculations of both correlation coefficients and *p*-values can be performed using the online statistics calculators available at http://www.danielsoper.com/statcalc3/. As a general comment, it should be noted that the confidence level of categorical data analysis increases greatly with the number of sequences in the expression datasets (Norman and Streiner, 2000).

## RESULTS AND DISCUSSION

Using the developed method, expression of 3066 human proteins and their domains in a cell-free bacterial system has been analyzed. It was found that the rate of soluble expression (score A) in the investigated dataset constituted 25.7% (Kurotani et al., 2010). This value should be considered as a benchmark, as the similar success rate has been reported for a different subset of human proteins expressed in *E. coli* (Ding et al., 2002). Furthermore, a number of statistically significant correlations between calculated and predicted properties of amino acid sequences and their amenability to bacterial cell-free expression have been identified using the developed approach. The most influential features that affect protein amenability to cell-free expression are listed in **Table 1**.

**Table 1:** Correlations of cell-free protein expression with calculated and predicted properties of amino acid sequences.

Expression property	Soluble	Insoluble	Undetectable
Length	–	ND	+
pI	±	±	ND
Charge	+	±	–
Hydrophobicity	–	+	ND
Solvent accessibility	+	–	+
Secondary structure	+	±	–
Intrinsic disorder	+	–	+
Protein domains	–	–	+
S–S bonds	–	+	+
Coiled coil	+	–	–
Transmembrane seqs	–	–	+
Localization signals	–	+	ND
PEST regions	+	–	+
Prenylation	+	ND	ND
Phosphorylation	+	–	–
Asn glycosylation	–	+	ND
Palmitoylation	–	±	+
Ubiquitination	+	–	ND
SUMOylation	+	–	±
Amidation	ND	ND	ND
Asx hydroxylation	ND	ND	ND
Sulfation	ND	ND	ND

*The signs (+) and (-) indicate positive and negative correlations, respectively; (±) refers to the opposite tendencies of expression estimates at different values of calculated parameters; and ND denotes the lack of correlation.*

Notably, some of these features, such as protein p*I*, hydrophobicity, presence of localization signals, etc., are mostly related to protein solubility, whereas the others, such as protein length, charge, solvent accessibility, presence of S–S bonds, transmembrane sequences, PEST regions, etc., also affect the overall expression propensity. The presence of some specific sequence motifs was found to be one of the most discriminative parameters for expression propensity. The correlations revealed can be of practical use for protein engineering with the aim of increasing expression success. The rationales for these correlations are discussed in detail in the published paper (Kurotani et al., 2010).

In addition, it was found that amenability of human polypeptide sequences to bacterial cell-free expression correlates with the presence of multiple PTM sites bioinformatically predicted in these sequences (Tokmakov et al., 2012; **Table 1**). Surprisingly, the presence of predicted sites for several PTMs, such as ubiquitination, SUMOylation, etc. (**Table 1**), was associated with increased production of properly folded soluble protein. However, no SUMOylation and ubiquitination machineries are known to exist in bacteria, suggesting that the presence of these PTM sites in amino acid sequences is related to intrinsically better protein solubility even in the absence of the modifications. It was hypothesized that physicochemical and/or structural characteristics of the modification sites themselves convey the better solubility (Tokmakov et al., 2012). Altogether, these findings indicate that identification of potential PTM sites in polypeptide sequences can be of practical use for predicting expression success and optimizing heterologous protein synthesis. Currently, a discriminant-based machine-learning algorithm that utilizes multiple features of amino acid sequences to predict the success rate of heterologous protein synthesis is being developed based on the reported findings. The algorithm will provide a basis for the internet-based tool for predicting amenability of eukaryotic proteins to cell-free expression in a prokaryotic system.

## Conflict of Interest Statement

The author declares that the research was conducted in the absence of any commercial or financial relationships that could be construed as a potential conflict of interest.

## Footnotes

1 http://www.expasy.org/tools/

2 http://scratch.proteomics.ics.uci.edu/explanation.html

3 http://mobyle.pasteur.fr/cgi-bin/portal.py?#forms::predator

4 http://emboss.sourceforge.net/apps/cvs/emboss/apps/pepcoil.html

5 http://www.strubi.ox.ac.uk/RONN

6 http://emboss.bioinformatics.nl/cgi-bin/emboss/pestfind

7 http://www.cbs.dtu.dk/services/SignalP/

8 http://harrier.nagahama-i-bio.ac.jp/sosui/

9 http://csspalm.biocuckoo.org
